# Lithium-Ion Transport and Exchange between Phases in a Concentrated Liquid Electrolyte Containing Lithium-Ion-Conducting Inorganic Particles

**DOI:** 10.1021/acsenergylett.4c00502

**Published:** 2024-03-25

**Authors:** Deyang Yu, Zachary C. Tronstad, Bryan D. McCloskey

**Affiliations:** † Energy Storage and Distributed Resources Division, Lawrence Berkeley National Laboratory, Berkeley, California 94720, United States; ‡ Department of Chemical & Biomolecular Engineering, University of California, Berkeley, California 94720, United States

## Abstract

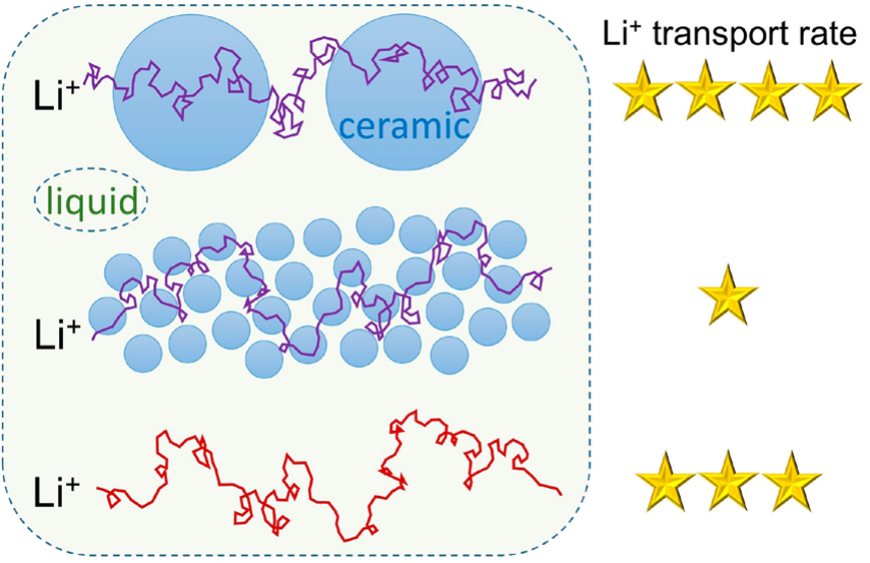

Understanding Li^+^ transport in organic–inorganic hybrid electrolytes, where Li^+^ has to lose its organic solvation shell to enter and transport through the inorganic phase, is crucial to the design of high-performance batteries. As a model system, we investigate a range of Li^+^-conducting particles suspended in a concentrated electrolyte. We show that large Li_1.3_Al_0.3_Ti_1.7_P_3_O_12_ and Li_6_PS_5_Cl particles can enhance the overall conductivity of the electrolyte. When studying impedance using a cell with a large cell constant, the Nyquist plot shows two semicircles: a high-frequency semicircle related to ion transport in the bulk of both phases and a medium-frequency semicircle attributed to Li^+^ transporting through the particle/liquid interfaces. Contrary to the high-frequency resistance, the medium-frequency resistance increases with particle content and shows a higher activation energy. Furthermore, we show that small particles, requiring Li^+^ to overcome particle/liquid interfaces more frequently, are less effective in facilitating Li^+^ transport. Overall, this study provides a straightforward approach to study the Li^+^ transport behavior in hybrid electrolytes.

In the development of all-solid-state lithium batteries, many Li^+^-conducting inorganic electrolytes have been studied, such as garnet Li_7_La_3_Zr_2_O_12_ (LLZO), NASICON-type Li_1.3_Al_0.3_Ti_1.7_P_3_O_12_ (LATP), perovskite Li_0.33_La_0.55_TiO_3_ (LLTO), and argyrodite Li_6_PS_5_Cl (LPSCl). These inorganic electrolytes typically have high Li^+^ conductivity (∼1 mS cm^–1^), high Li^+^ transference number (∼1), excellent thermal stability, and wide temperature capability.^1−7^ However, when these inorganic electrolyte particles are compressed and sintered into pellets, the pellets are generally very brittle, leading to poor electrolyte/electrode contact and a high resistance.^8^ The voids between particles are vulnerable to lithium dendrites, leading to potential short-circuiting.^9−11^ Inorganic electrolytes often have low (electro)chemical stability against the cathodes or anodes.^12−14^ Liquid electrolytes and/or polymer electrolytes are thus commonly combined in use with inorganic electrolytes to compensate for the above issues and improve electrolyte and electrode processability.^15−17^ Li^+^ transport dynamics crossing the interfaces between the hard inorganic electrolyte and the soft liquid or polymer electrolyte, accompanying the Li^+^ (de)solvation processes, is thus crucial to the overall performance of a lithium cell.

In a hybrid electrolyte composed of a soft liquid or polymer electrolyte matrix with dispersed hard inorganic solid electrolyte particles, the potential Li^+^ transport pathways are (1) entirely through the soft matrix (i.e., without ever entering an inorganic particle), (2) along inorganic particle surfaces through percolating particle networks,^18−21^ and (3) through both the inorganic particles and the soft phases by transferring through the soft/hard interfaces (Figure 1a).^22−25^ Li^+^ may even encounter some combination of these transport modes. In addition to the discussion of the Li^+^ transport mechanism, a primary question is whether the addition of inorganic particles to the soft matrix can improve the conductivity of the electrolyte. It is well known that adding a small fraction of inorganic particles into a polymer electrolyte can effectively suppress crystallization of the polymer phase and thus benefit Li^+^ transport in the polymer matrix. However, a continuous increase of the conductivity with increasing inorganic particle content is generally not observed.^26−30^ Some studies further showed that the incorporation of inorganic electrolyte particles does little to help the conductivity of the polymer matrix.^31,32^ Using model liquid electrolytes, Isaac et al. found that porous Li^+^-conducting particles act as insulators, whereas dense particles act as conductors.^33^

**Figure 1:**
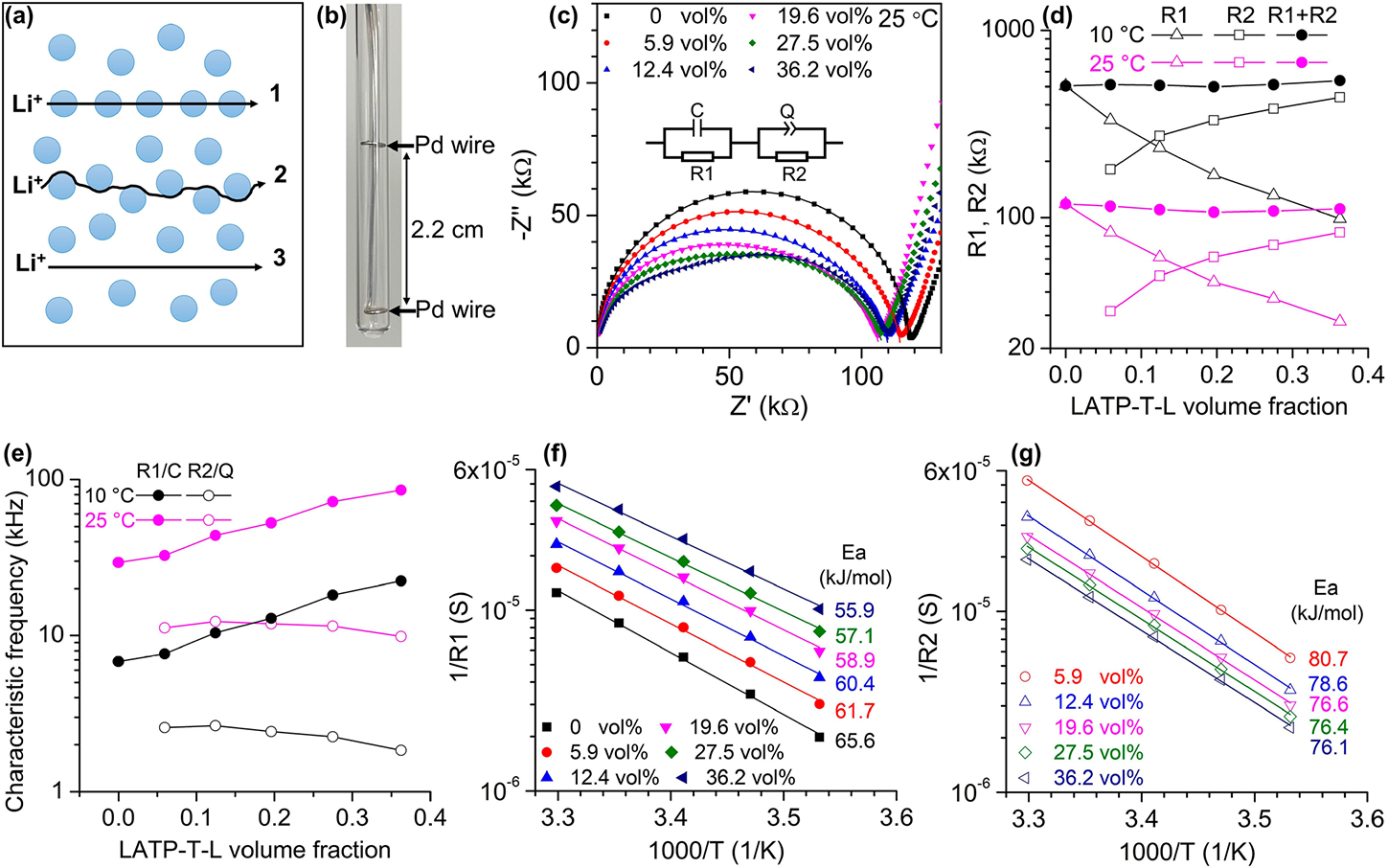
Impedance study of LATP-T-L particles suspended in a high-concentration liquid electrolyte composed of EC and LiTFSI in a 2/1 molar ratio. (**a**) Schematic illustration of the three potential Li^+^ transport pathways: through inorganic particles (1), along the particle surface (2), and through the liquid phase only (3). (**b**) An image of the cell used for all the impedance measurements in this study. Two Pd spiral wires serving as electrodes are separated by 2.2 cm, and the cell constant is calibrated using KCl standard solution to be 19.5 cm^–1^. The error of conductivity measurements using this cell is estimated to be <±10%. (**c**) Nyquist plots of LATP-T-L particles suspended in EC/LiTFSI = 2/1 at 25 °C. The content of LATP particles in the suspension ranges from 0 to 36 vol% (0–50 wt%). The solid lines are fits to the experimental data points before electrode polarization using the equivalent circuit shown in the inset. (**d**) Variations of R1 and R2 as a function of the LATP volume fraction at 25 and 10 °C. (**e**) Characteristic frequencies of the R1 and R2 processes obtained from impedance fitting at 25 and 10 °C. Arrhenius plots of the inverse of R1 (**f**) and the inverse of R2 (**g**) from 10 to 30 °C, along with their activation energies.

One of the reasons this understanding is currently vague is the lack of tools available for studying the Li^+^ transport mechanism in hybrid electrolytes. The current tools typically include lithium 2D exchange spectroscopy (EXSY, to evaluate chemical exchange of Li^+^ in different environments) and a ^6^Li/^7^Li isotope replacement method.^19,24,30,31,34−36^ The main method to evaluate the resistance of Li^+^ transporting through the soft/hard interfaces is to build soft/hard/soft trilayer sandwiched electrolyte structures and then measure the impedance of the sandwich.^37−40^ In this study, we show that valuable information about ion transport mechanisms can be obtained simply by studying suspensions of inorganic particles in a high-concentration liquid electrolyte. We believe the procedures established here could be easily extended to polymer–inorganic hybrids as well. We also demonstrate that particle size plays a crucial role in determining the ion transport behavior in hybrid electrolytes, which could help unite the various disparate conclusions described in the aforementioned studies.

Although the Li^+^ conductivity of inorganic electrolytes can be almost as high as that of liquid electrolytes, the mobility of individual Li^+^ is still much lower than that in commercial liquid electrolytes, because Li^+^ concentration in inorganic electrolytes is generally much higher. For example, the concentration of Li^+^ in LPSCl is up to 36.7 mol dm^–3^, more than 1 order of magnitude higher than that in commercial liquid electrolytes. In order to magnify the contribution of inorganic particles to Li^+^ transport, the mobility of Li^+^ in the liquid phase should be comparable to or lower than the Li^+^ mobility inside the particles. The ionic conductivity of a high-concentration liquid electrolyte prepared by dissolving lithium bis(trifluoromethylsulfonyl)imide (LiTFSI) in ethylene carbonate (EC) with a molar ratio of EC/LiTFSI = 2/1 has been measured to be ∼0.1 mS cm^–1^ at 20 °C and ∼0.25 mS cm^–1^ at 30 °C,^41^ comparable to the ionic conductivity values of polymer electrolytes and much lower than those of most of the inorganic electrolytes studied here. Thus, we choose EC/LiTFSI = 2/1 as a starting high-concentration electrolyte to examine whether adding inorganic electrolyte particles can increase its conductivity. Previous simulation studies by Kim et al. indicated that the particle sizes of inorganic electrolytes can affect the conductivity of composite electrolytes.^42^ Thus, in addition to the chemical structure of inorganic electrolytes, we also examine the effect of their particle sizes on the conductivity of their suspensions in EC/LiTFSI = 2/1. The various inorganic particles used in this study, along with the vendors, particle sizes, and conductivities, are summarized in Table 1. SEM images of these particles are shown in Figures S1–S3. We also note that, due to the high liquid electrolyte viscosity, all suspensions were stable over at least multiple hours, if not days, allowing good impedance reproducibility. (See Figure S4 for an example using LPSCl-M-L particles.)

**Table 1:** Chemical Composition, Vendor, Particle Size, and Conductivity of the Inorganic Electrolytes Used in This Study

Inorganic electrolyte^a^	Chemical Composition	Vendor	Particle Size D50 (μm)^b^	Conductivity (mS cm^–1^)^c^
LATP-T-L	Li_1.3_Al_0.3_Ti_1.7_P_3_O_12_	Toshima Manufacturing	13	0.7^b^
LATP-T-S	Li_1.3_Al_0.3_Ti_1.7_P_3_O_12_	Toshima Manufacturing	1	0.7^b^
LATP-M-S	Li_1.3_Al_0.3_Ti_1.7_P_3_O_12_	MSE Supplies	0.6	0.6–0.8^b^
LPSCl-M-L	Li_6_PS_5_Cl	MSE Supplies	∼80	2–5^b^
LPSCl-M-M	Li_6_PS_5_Cl	MSE Supplies	∼10	1–4^b^
LPSCl-M-S	Li_6_PS_5_Cl	MSE Supplies	∼1	≥0.1^b^
LLTO-T-L	Li_0.33_La_0.55_TiO_3_	Toshima Manufacturing	8	1.1^6^
LLTO-T-S	Li_0.33_La_0.55_TiO_3_	Toshima Manufacturing	0.4	1.1^6^
Ta-LLZO-T-L	Li_6.6_La_3_Zr_1.6_Ta_0.4_O_12_	Toshima Manufacturing	6	1.2^43^
LICGC-O-S	Li_1+*x*+*y*_Al_*x*_Ti_2–*x*_Si_*y*_P_3–*y*_O_12_	Ohara	0.9	∼1^b^

a The first letter after the short name of each inorganic electrolyte represents the vendor (T for Toshima Manufacturing, M for MSE Supplies, and O for Ohara), and the last letter denotes the particle size (L for large particle size, M for medium size, and S for small size).

b Provided by vendors.

c The conductivity of LLTO is its bulk conductivity, and the conductivities for all other samples are total conductivities.

In impedance studies, when the frequency of the applied AC potential is low, ions are blocked at the electrode/electrolyte interface and cause electrode polarization. Increasing the distance between electrodes shifts electrode polarization to even lower frequencies.^44−46^Figure 1b shows a lab-built cell used to measure the conductivity of the suspensions. The electrodes are separated by 2.2 cm, and the cell constant is calibrated to be 19.5 cm^–1^. Due to the large distance between electrodes, the onset of electrode polarization is measured to be ∼1 kHz for the neat liquid electrolyte EC/LiTFSI = 2/1 at 25 °C, enabling the observance of a complete and standard semicircle in the Nyquist plot (Figure 1c, black squares), which in this single-phase electrolyte corresponds to its bulk resistance, as subsequently explained. A standard R/C circuit (a resistor in parallel with a capacitor) fits the experimental data very well, giving a bulk resistance of 118 kΩ and correspondingly a conductivity of 0.165 mS cm^–1^ for the neat liquid electrolyte at 25 °C via the following equation relating the bulk resistance (*R*, kΩ), cell constant (*k*, cm^–1^), and electrolyte conductivity (σ, mS cm^–1^):
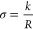
1The electrolyte conductivity agrees very well with previously reported values,^41^ confirming that the R/C semicircle in the Nyquist plot is related to the bulk resistance of the electrolyte.

After addition of relatively large diameter (D50 = 13 μm) LATP-T-L particles into the liquid electrolyte, the semicircle is suppressed and can be well-fit as two processes: a high-frequency R/C circuit in series with a medium-frequency R/Q circuit (a resistor in parallel with a constant-phase element) (Figure 1c). The fitted curve (solid lines in Figure 1c) agrees with the semicircle portions of the experimental data surprisingly well, and final fitting parameters are shown in Table S1. The high-frequency resistance (R1) decreases drastically with increasing LATP-T-L content, while the medium-frequency resistance (R2) increases with the particle content (Figure 1d). It is interesting that the overall resistance, i.e., the intersection of the low-frequency straight line and the semicircle, of the suspensions is simply the sum of a high-frequency resistance R1 and a medium-frequency resistance R2. The lowest overall resistance is observed to be 106 kΩ at 19.6 vol% particle content, leading to an increase of the conductivity by 11% compared to that of the neat liquid electrolyte. It is clear that the increase of R2 is the main issue preventing the suspensions from reaching a much higher conductivity when the particle content is increased.

Figure 1e shows the characteristic frequencies of the R1 and R2 processes calculated from the impedance fitting parameters. The characteristic frequency of the R1 process, which is 1/(2πR1C), increases with LATP-T-L particle content by ×3 from 0 to 36 vol% particle content, indicating that R1 is not solely determined by the liquid electrolyte. Because the bulk conductivity of LATP is up to 3–5 mS cm^–1^ with a characteristic frequency >1 MHz,^32,47,48^ it is likely that the Li^+^ motion inside LATP particles affects the R1 process, leading to both a decrease of R1 resistance and an increase of its characteristic frequency when LATP content in the suspension is increased. In contrast, the characteristic frequency of the R2 process shows only a slight decrease with increasing LATP content, suggesting that the process associated with R2 is fairly independent of inorganic particle volume fraction.

The temperature dependences of R1 and R2 are shown in Figure 1f,g. Within a narrow temperature window, 10–30 °C, the Arrhenius equation fits the temperature dependence of the inverse of R1 (1/R1) and the inverse of R2 (1/R2) very well, and the activation energies for these two processes at various particle contents are obtained. For the neat high-concentration liquid electrolyte, the activation energy for its ionic conductivity is 65.6 kJ mol^–1^. Adding LATP-T-L particles leads to a significant decrease in the activation energy for the R1 process, again indicating that the relatively high mobility of Li^+^ inside LATP particles is affecting this high-frequency process. The activation energy for the medium-frequency process R2 also decreases with increasing LATP-T-L contents. However, the activation energy for the medium-frequency process is much higher than that for the high-frequency process. If ions in the suspensions prefer to transport along the particle surface as shown in Figure 1a, path 2, it is hard to imagine that this process could increase conductivity yet have a higher activation energy than ions transporting in the neat liquid electrolyte. Thus, we attribute the medium-frequency process R2 to Li^+^ transporting through the particle/liquid interface, and we attribute the high-frequency process R1 to ion transport in the bulk of both the liquid phase and LATP particles. Note that, if we assume the conductivity of the liquid phase does not change after addition of the particles, the drastic decrease of R1 with increasing LATP-T-L content is beyond the prediction of the Maxwell effective medium theory.^33,49^

In order to further analyze the effect of particle size on the ionic conductivity of suspensions, LATP particles with smaller sizes (LATP-M-S with 0.6 μm average diameter and LATP-T-S with 1 μm average diameter) are examined. The Nyquist plots of EC/LiTFSI = 2/1 with 12 vol% LATP-T-S and LATP-M-S are shown in Figure 2a. In contrast to the previous samples with large particles, no obvious R2 process is observed for these two suspensions, although the plot of LATP-T-S slightly deviates from a standard single semicircle. The characteristic frequencies of these two electrolytes are very close to that of the neat liquid electrolyte (22–28 kHz), whereas the R1 characteristic frequency for the 12 vol% LATP-T-L is substantially higher (44 kHz). In addition, both of these smaller LATP particles decrease the conductivity of the high-concentration electrolyte by ∼20% at 12 vol% LATP, strongly suggesting that these smaller LATP particles behave like Li^+^-insulators.

**Figure 2:**
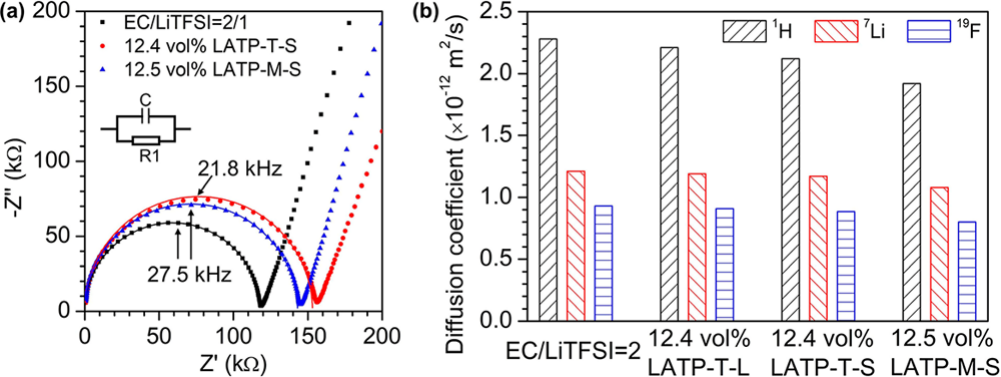
Effect of LATP particle size on ion transport. (**a**) Nyquist plots of 12 vol% LATP-T-S and LATP-M-S in EC/LiTFSI = 2/1 suspensions at 25 °C. The plot of the neat EC/LiTFSI is also shown as a comparison. The plots are fitted with a resistor in parallel with a capacitor. (**b**) Diffusion coefficients of EC (^1^H), Li^+^ (^7^Li), and TFSI^–^ (^19^F) in the high-concentration liquid electrolyte and suspensions with 12 vol% LATP at 25 °C. The standard deviation of the diffusion coefficient measurements is estimated to be within ±5%.

We further measure the diffusion coefficients of the ions and solvent molecules in the liquid phase using pulsed-field-gradient nuclear magnetic resonance (PFG-NMR; see the Supporting Information for more details). Figure 2b shows the diffusion coefficients of the neat high-concentration electrolyte and the suspensions with 12 vol% LATP particles. Adding 12 vol% LATP leads to only slight decreases of the diffusion coefficients of all the species (EC, Li^+^, and TFSI^–^) uniformly in the liquid phase. In addition, no significant differences are observed for the suspensions with different LATP particles. This indicates that LATP particles do not have preferential interactions with Li^+^, agreeing with our previous conclusion that the R2 process is not related to Li^+^ transport along the particle surface.

A potential explanation for why the small LATP particles behave like Li^+^-insulators is that their surface is covered by a thin layer of an impurity (e.g., lithium carbonate or phosphate), blocking Li^+^ transport. Instead of conducting surface analysis, such as X-ray photoelectron spectroscopy, here we present a simple but robust Li^+^/Na^+^ exchange experiment to show that the Li^+^ transporting pathways on the particle surface are not blocked. In this experiment, we mix LATP particles with a NaClO_4_/EC solution for a certain time. The particles are then filtered out using a syringe filter, and the concentrations of Na^+^ and Li^+^ in the obtained clear solution are analyzed using NMR with an external standard (see the Supporting Information for more details). Figure 3 shows the ^7^Li and ^23^Na NMR spectra of the solution after 5 min, 15 min, 1 h, and 20 h mixing for two LATP materials, LATP-T-L and LATP-M-S. Upon increasing the mixing time of LATP-T-L and the NaClO_4_/EC solution, we see an increase of the ^7^Li signal intensity and a decrease of the ^23^Na signal intensity, while the total concentration of Li^+^ and Na^+^ in the clear solution remains almost constant. This indicates that Li^+^ inside the LATP-T-L particles and Na^+^ in the solution are slowly exchanging with each other. When using LATP-M-S particles, the intensity of the ^7^Li signal is substantial even after just 5 min of mixing and reaches an equilibrium after about 15 min. Figure 3c shows the molar ratio of Li^+^/(Li^+^ + Na^+^) in the clear solution after Li^+^/Na^+^ exchange. It clearly shows that the Li^+^/Na^+^ exchange between small particles and the liquid solution is much faster.

**Figure 3:**
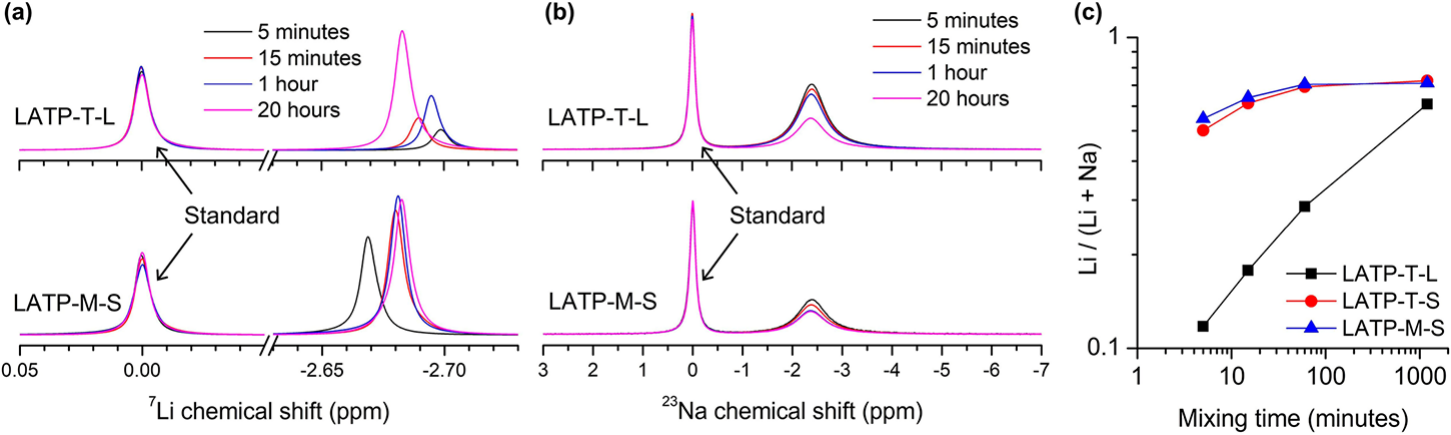
A Li^+^/Na^+^ exchange experiment. After mixing of LATP particles with a NaClO_4_/EC solution (the molar ratio between Li^+^ inside LATP and Na^+^ in the solution is controlled to 1:1) for a certain time, the particles are filtered out and ^7^Li NMR (**a**) and ^23^Na NMR (**b**) spectra of the clear solution are recorded. The upper panels are the spectra when using LATP-T-L particles, and the lower panels correspond to LATP-M-S particles. An external standard composed of 0.5 M NaClO_4_ and 0.5 M LiTFSI in acetone is used to measure the concentrations of Na^+^ and Li^+^ in the solution. (**c**) Molar ratio of Li^+^ in the solution after Li^+^/Na^+^ exchange as a function of mixing time.

First instinct suggests that the Li^+^/Na^+^ exchanging result “contradicts” the impedance results, because the impedance measurements indicate that small LATP particles behave like Li^+^-insulators, while the Li^+^/Na^+^ exchange experiment claims the ion exchange with small LATP particles is faster. In fact, the faster ion exchange between small LATP particles and the liquid electrolyte is mainly because of the much larger specific surface area of the smaller particles. If we assume the particles are perfect spheres, then the specific surface area (total surface area per unit mass of LATP) would be inversely proportional to the particle size. Since the particle sizes of the small LATP are about an order of magnitude smaller than those of the large particles (Table 1), the total surface area where the ion-exchange process happens is thus an order of magnitude larger, leading to 10× more ions involved in the Li^+^/Na^+^ exchange process at the same time. It is important to note that, although the Li^+^/Na^+^ exchange is fast for the small LATP particles, a time scale of ∼ 1 min is still needed in order to observe substantial Li^+^/Na^+^ exchange. The time scale for the impedance measurements, however, is below 1 ms (corresponding to an AC frequency of >1 kHz). In addition, the Li^+^/Na^+^ exchange is a static ion exchange without any external electric field, while the impedance measures the collective migration of ions in an external electric field. We also note that the molar ratio of Li^+^/(Li^+^ + Na^+^) in the clear solution is >0.5 at equilibrium, although the molar ratio of Li^+^ and Na^+^ in the system is controlled to 1:1, indicating Li^+^ has a higher solvation energy in the solution compared to Na^+^. Overall, the Li^+^/Na^+^ exchange experiment clearly indicates that the ion transport pathways on the small LATP particle surfaces are not blocked. We have also measured the content of Li_2_CO_3_ on the surface of LATP particles using titration mass spectrometry,^50^ and only a trace amount (<0.01%) of Li_2_CO_3_ is found.

In order to further demonstrate that the size of inorganic electrolyte particles can greatly affect the overall conductivity of composite electrolytes, we measure the impedance of suspensions prepared with three LPSCl materials provided from the same vendor. Large LPSCl particles (D50 ≈ 80 μm) greatly improve the conductivity of the high-concentration electrolyte EC/LiTFSI = 2/1. As shown in Figure 4a, adding 36 vol% LPSCl-M-L into the liquid electrolyte reduces the overall resistance to 65.3 kΩ, leading to an overall conductivity of 0.299 mS cm^–1^, which is 1.8× that of the neat liquid electrolyte. In contrast, the suspensions with medium and small LPSCl particles (blue and red data in Figure 4a) have lower conductivities than the pure liquid electrolyte, although two overlapping semicircles are still present in these Nyquist plots, suggesting that the particles do not act as Li^+^-insulators, as is the case with the small LATP particles (Figure 2).

**Figure 4:**
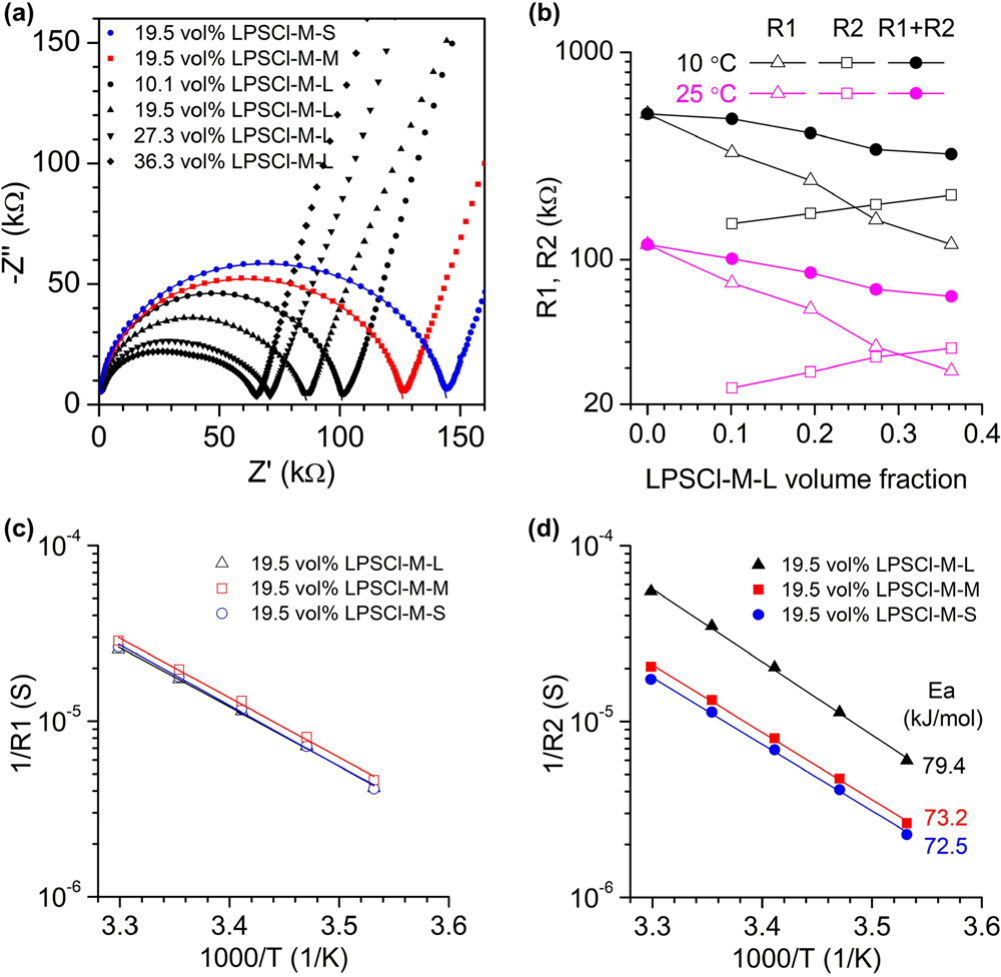
Impedance study of LPSCl suspended in EC/LiTFSI = 2/1. (**a**) Nyquist plots of the suspensions with LPSCl-M-S (blue), LPSCl-M-M (red), and LPSCl-M-L (black) particles at 25 °C. The plots are fitted with the same equivalent circuit as shown in Figure 1c. (**b**) Variations of R1, R2, and the sum of R1 and R2 as a function of LPSCl-M-L volume fraction at 25 and 10 °C. Arrhenius plots of 1/R1 (**c**) and 1/R2 (**d**) of the suspensions with the same amount of LPSCl particles but varying particle sizes.

The Nyquist plots of all the suspensions with LPSCl particles are fitted with the R1/C + R2/Q equivalent circuit. As in the case of LATP-T-L, R1 decreases and R2 increases when the content of LPSCl is increased (Figure 4b). The high-frequency resistance values R1 of suspensions with different LPSCl particles at the same particle content are nearly identical, regardless of the temperature (Figure 4c), with an activation energy of 64–66 kJ mol^–1^. The medium-frequency resistance R2 is significantly affected by the particle size. Reducing the LPSCl particle size causes a substantial increase in R2. The activation energy for the R2 process is 72–79 kJ mol^–1^, again much higher than that for the R1 process. The activation energy for the large LPSCl R2 (79.4 kJ mol^–1^) is similar to the R2 activation energies observed in the large LATP particles (Figure 1g, 76.6 kJ/mol^–1^) at similar particle loadings, suggesting that the R2 process is controlled by the liquid electrolyte properties, particularly the desolvation of Li^+^. The activation energies for LPSCl R1 (64–66 kJ mol^–1^) are slightly larger than the LATP R1 values at a similar loading (Figure 1f, 58.9 kJ mol^–1^ at 19.6 vol% loading), further suggesting that ion transport through the bulk of each inorganic particle contributes to the R1 value.

We further examine the rest of the inorganic electrolyte particles listed in Table 1. Among them, only the suspensions with LLTO-T-L particles demonstrate medium-frequency resistance (Figure S5). However, the R2 resistance is much higher than that in the suspensions with LATP-T-L particles at the same conditions, leading to a decrease of the overall conductivity of the suspensions with increasing LLTO-T-L content. However, the major trends of R1 and R2 stay the same, such as R1 decreasing and R2 increasing with particle content and the R2 process demonstrating a higher activation energy than the R1 process. For all the other inorganic electrolytes, the Nyquist plots show only one semicircle (Figure S6). We note in passing that the Ta-LLZO-T-L sample appeared to react with the liquid electrolyte and dramatically increase its viscosity (from visual inspection), hence the observation of a very large Nyquist semicircle compared to all other electrolytes studied.

From the above study, we can bin the behavior of the studied inorganic electrolyte particles into two categories. The first category includes LATP-T-S, LATP-M-S, LICGC-O-S, and LLTO-T-S. When these small inorganic electrolyte particles are dispersed in the high-concentration liquid electrolyte EC/LiTFSI = 2/1, their Nyquist plots shows only one semicircle, just like the neat liquid electrolyte, and the conductivities of these suspensions are very close to each other at the same particle volume fraction (Figure S7). The second category consists of LATP-T-L, LLTO-T-L, and the three LPSCl materials. The Nyquist plots of suspensions with these materials demonstrate a high-frequency semicircle and a medium-frequency semicircle. Increasing the particle content leads to a decrease of the high-frequency resistance R1 and an increase of the medium-frequency resistance R2. Reducing the particle size increases R2 as well. The conductivities of these suspensions are no less than those of the first category. The Li^+^/Na^+^ exchange experiment indicates that the surface chemistry (in particular, the presence of insulating impurities) is not responsible for the differences between these two categories.

Instead, our results suggest that the size of the particles is a major reason for the differences between the two categories of particles. When Li^+^ has to overcome a high energy barrier in order to transport through the inorganic particles, larger particles can potentially allow the Li^+^ to migrate smoothly for a longer distance once it traverses the particle/liquid interface. If we consider only migration, the number of interfaces a Li^+^ needs to transport through is inversely proportional to the particle size in order to migrate inside particles for the same length (Figure 5, pathways **a** and **a′**). In addition to migration, ions also diffuse randomly due to thermal motion, which is often not highlighted in the discussion of conductivity in literature. When considering random diffusion, Li^+^ likely needs to transport through more interfaces in order to demonstrate the same migration length, as illustrated in pathways **b** and **b′** in Figure 5. Thus, random diffusion further amplifies the difference between the large particles and small particles. In fact, by comparing the root-mean-square displacement () of Li^+^ caused by diffusion and its migration length (μ*Et*) caused by an external electric field, we can easily find that diffusion dominates the motions of Li^+^ on a length scale within a few micrometers for typical electric fields felt in a battery (Figure S8). The motion of Li^+^ is nearly identical to a random walk within such a length scale in the high-concentration electrolyte. Thus, the effect of diffusion on Li^+^ transport is far more dominant even than the schematic illustration in Figure 5 shows. It is probably even possible that the same Li^+^ needs to transport through the same small particle multiple times before this Li^+^ demonstrates a significant migration length. For the suspensions with small particles, it is much easier for Li^+^ to diffuse around the particles via the liquid phase rather than transporting through it when a high energy barrier exists at the particle/liquid interface. Thus, Li^+^ transporting through small particles contributes little to the overall measured current signal in an impedance measurement relative to Li^+^ transporting solely through the liquid phase, especially when the volume fraction of particles is not very high, as in this study, and thus the medium-frequency semicircle is not detected for the “first category” (small) particles in EC/LiTFSI = 2/1.

**Figure 5:**
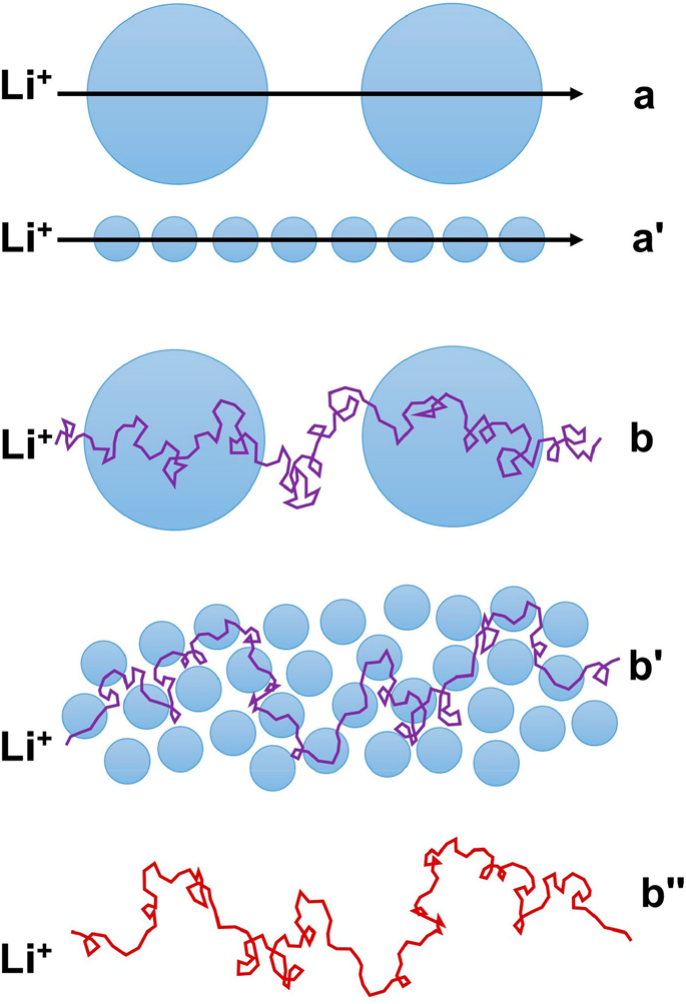
Schematic illustration of the particle size effect on ion transport. When it is inevitable to overcome a high energy barrier at the particle/liquid interface, small particles require Li^+^ to transport through more interfaces in order to migrate for the same length (pathways **a** and **a′**). Diffusion of Li^+^ further amplifies the difference between large particles and small particles (pathways **b** and **b′**), requiring Li^+^ to transport through even more interfaces, ultimately leading to a negligible contribution to the current signal measured in an impedance experiment relative to the contribution from ions transporting in the liquid phase only (pathway **b″**). The ratio of the diameters of the large circle and small circle shown in this figure is only 4:1.

In this study, we investigated the suspensions of solid inorganic Li^+^-conducting particles in high-concentration solutions to understand ion transport across soft–hard interfaces that require ion desolvation. After addition of large LATP particles or LPSCl particles to EC/LiTFSI = 2/1, contributions to the overall resistance of the suspensions include a high-frequency resistance and a medium-frequency resistance. The high-frequency resistance is dominated by the liquid phase, although increasing the inorganic particle content decreases this high-frequency resistance. We attribute the medium-frequency resistance to Li^+^ transporting through particle/liquid interfaces. This resistance increases with particle content and demonstrates a higher activation energy, indicating that higher temperatures will more dramatically impact interfacial transport in hybrid electrolytes. Our study indicates that the particle size is very important to the conductivity of hybrid electrolytes. Larger particle sizes allow Li^+^ to migrate over longer distances within high-conductivity inorganic particles without crossing a large number of high-activation-energy particle/liquid interfaces. In fact, we find that solid Li^+^-conducting particles with small average diameters (<1 μm) act as Li^+^-insulators in most cases, even though our Li^+^/Na^+^ exchange experiments suggest that the small particles remain active for static ion exchange with the liquid electrolyte. Thus, using inorganic particles with a diameter close to the targeted electrolyte thickness is strongly suggested when preparing solid-state composite electrolytes.

## Data Availability

All data supporting the findings of this study are available from the corresponding author upon reasonable request.

## Supporting Information Available

Experimental section, fitting parameters for the Nyquist plots, SEM images of the inorganic particles, Nyquist plots of suspensions with LLTO-T-L particles and the temperature dependence of 1/R1 and 1/R2, Nyquist plots of suspensions with other inorganic particles, Arrhenius plots of the conductivity, comparison between the diffusion length and migration length for the high-concentration electrolyte, and stability test of the impedance measurements (PDF)

## References

[ref1] Murugan R.; Thangadurai V.; Weppner W. Fast Lithium Ion Conduction in Garnet-Type Li_7_La_3_Zr_2_O_12_. Angew. Chem., Int. Ed. 2007, 46 (41), 7778–7781. 10.1002/anie.200701144. 17803180

[ref2] Aono H.; Sugimoto E.; Sadaoka Y.; Imanaka N.; Adachi G. y. Ionic Conductivity of Solid Electrolytes Based on Lithium Titanium Phosphate. J. Electrochem. Soc. 1990, 137 (4), 1023 10.1149/1.2086597.

[ref3] Deiseroth H.-J.; Kong S.-T.; Eckert H.; Vannahme J.; Reiner C.; Zaiß T.; Schlosser M. Li_6_PS_5_X: A Class of Crystalline Li-Rich Solids With an Unusually High Li^+^ Mobility. Angew. Chem., Int. Ed. 2008, 47 (4), 755–758. 10.1002/anie.200703900. 18161703

[ref4] Bachman J. C.; Muy S.; Grimaud A.; Chang H.-H.; Pour N.; Lux S. F.; Paschos O.; Maglia F.; Lupart S.; Lamp P.; Giordano L.; Shao-Horn Y. Inorganic Solid-State Electrolytes for Lithium Batteries: Mechanisms and Properties Governing Ion Conduction. Chem. Rev. 2016, 116 (1), 140–162. 10.1021/acs.chemrev.5b00563. 26713396

[ref5] Inaguma Y.; Liquan C.; Itoh M.; Nakamura T.; Uchida T.; Ikuta H.; Wakihara M. High ionic conductivity in lithium lanthanum titanate. Solid State Commun. 1993, 86 (10), 689–693. 10.1016/0038-1098(93)90841-A.

[ref6] Kawai H.; Kuwano J. Lithium Ion Conductivity of A-Site Deficient Perovskite Solid Solution La_0.67–x_Li_3x_TiO_3_. J. Electrochem. Soc. 1994, 141 (7), L78 10.1149/1.2055043.

[ref7] Famprikis T.; Canepa P.; Dawson J. A.; Islam M. S.; Masquelier C. Fundamentals of inorganic solid-state electrolytes for batteries. Nat. Mater. 2019, 18 (12), 1278–1291. 10.1038/s41563-019-0431-3. 31427742

[ref8] Pervez S. A.; Cambaz M. A.; Thangadurai V.; Fichtner M. Interface in Solid-State Lithium Battery: Challenges, Progress, and Outlook. ACS Appl. Mater. Interfaces 2019, 11 (25), 22029–22050. 10.1021/acsami.9b02675. 31144798

[ref9] Janek J.; Zeier W. G. A solid future for battery development. Nat. Energy 2016, 1 (9), 16141 10.1038/nenergy.2016.141.

[ref10] Dixit M. B.; Singh N.; Horwath J. P.; Shevchenko P. D.; Jones M.; Stach E. A.; Arthur T. S.; Hatzell K. B. In Situ Investigation of Chemomechanical Effects in Thiophosphate Solid Electrolytes. Matter 2020, 3 (6), 2138–2159. 10.1016/j.matt.2020.09.018.

[ref11] Singh D. K.; Henss A.; Mogwitz B.; Gautam A.; Horn J.; Krauskopf T.; Burkhardt S.; Sann J.; Richter F. H.; Janek J. Li_6_PS_5_Cl microstructure and influence on dendrite growth in solid-state batteries with lithium metal anode. Cell Rep. Phys. Sci. 2022, 3 (9), 101043 10.1016/j.xcrp.2022.101043.

[ref12] Dewald G. F.; Ohno S.; Kraft M. A.; Koerver R.; Till P.; Vargas-Barbosa N. M.; Janek J.; Zeier W. G. Experimental Assessment of the Practical Oxidative Stability of Lithium Thiophosphate Solid Electrolytes. Chem. Mater. 2019, 31 (20), 8328–8337. 10.1021/acs.chemmater.9b01550.

[ref13] Schwietert T. K.; Arszelewska V. A.; Wang C.; Yu C.; Vasileiadis A.; de Klerk N. J. J.; Hageman J.; Hupfer T.; Kerkamm I.; Xu Y.; van der Maas E.; Kelder E. M.; Ganapathy S.; Wagemaker M. Clarifying the relationship between redox activity and electrochemical stability in solid electrolytes. Nat. Mater. 2020, 19 (4), 428–435. 10.1038/s41563-019-0576-0. 31932670

[ref14] Thompson T.; Yu S.; Williams L.; Schmidt R. D.; Garcia-Mendez R.; Wolfenstine J.; Allen J. L.; Kioupakis E.; Siegel D. J.; Sakamoto J. Electrochemical Window of the Li-Ion Solid Electrolyte Li_7_La_3_Zr_2_O_12_. ACS Energy Lett. 2017, 2 (2), 462–468. 10.1021/acsenergylett.6b00593.

[ref15] Woolley H. M.; Vargas-Barbosa N. M. Hybrid solid electrolyte-liquid electrolyte systems for (almost) solid-state batteries: Why, how, and where to?. J. Mater. Chem. A 2023, 11 (3), 1083–1097. 10.1039/D2TA02179J.

[ref16] Keller M.; Varzi A.; Passerini S. Hybrid electrolytes for lithium metal batteries. J. Power Sources 2018, 392, 206–225. 10.1016/j.jpowsour.2018.04.099.

[ref17] Tang S.; Guo W.; Fu Y. Advances in Composite Polymer Electrolytes for Lithium Batteries and Beyond. Adv. Energy Mater. 2021, 11 (2), 2000802 10.1002/aenm.202000802.

[ref18] Zheng J.; Wang P.; Liu H.; Hu Y.-Y. Interface-Enabled Ion Conduction in Li_10_GeP_2_S_12_–Poly(ethylene Oxide) Hybrid Electrolytes. ACS Appl. Energy Mater. 2019, 2 (2), 1452–1459. 10.1021/acsaem.8b02008.

[ref19] Yan Y.; Ju J.; Dong S.; Wang Y.; Huang L.; Cui L.; Jiang F.; Wang Q.; Zhang Y.; Cui G. In Situ Polymerization Permeated Three-Dimensional Li^+^-Percolated Porous Oxide Ceramic Framework Boosting All Solid-State Lithium Metal Battery. Adv. Sci. 2021, 8 (9), 2003887 10.1002/advs.202003887. PMC8097327 33977057

[ref20] Zaman W.; Hortance N.; Dixit M. B.; De Andrade V.; Hatzell K. B. Visualizing percolation and ion transport in hybrid solid electrolytes for Li–metal batteries. J. Mater. Chem. A 2019, 7 (41), 23914–23921. 10.1039/C9TA05118J.

[ref21] Yang H.; Abdullah M.; Bright J.; Hu W.; Kittilstved K.; Xu Y.; Wang C.; Zhang X.; Wu N. Polymer-ceramic composite electrolytes for all-solid-state lithium batteries: Ionic conductivity and chemical interaction enhanced by oxygen vacancy in ceramic nanofibers. J. Power Sources 2021, 495, 229796 10.1016/j.jpowsour.2021.229796.

[ref22] Fu J.; Li Z.; Zhou X.; Guo X. Ion transport in composite polymer electrolytes. Mater. Adv. 2022, 3 (9), 3809–3819. 10.1039/D2MA00215A.

[ref23] Horowitz Y.; Lifshitz M.; Greenbaum A.; Feldman Y.; Greenbaum S.; Sokolov A. P.; Golodnitsky D. Review—Polymer/Ceramic Interface Barriers: The Fundamental Challenge for Advancing Composite Solid Electrolytes for Li-Ion Batteries. J. Electrochem. Soc. 2020, 167 (16), 160514 10.1149/1945-7111/abcd12.

[ref24] Nkosi F. P.; Valvo M.; Mindemark J.; Dzulkurnain N. A.; Hernández G.; Mahun A.; Abbrent S.; Brus J.; Kobera L.; Edström K. Garnet-Poly(ε-caprolactone-co-trimethylene carbonate) Polymer-in-Ceramic Composite Electrolyte for All-Solid-State Lithium-Ion Batteries. ACS Appl. Energy Mater. 2021, 4 (3), 2531–2542. 10.1021/acsaem.0c03098.

[ref25] Li Z.; Fu J.; Zhou X.; Gui S.; Wei L.; Yang H.; Li H.; Guo X. Ionic Conduction in Polymer-Based Solid Electrolytes. Adv. Sci. 2023, 10 (10), 2201718 10.1002/advs.202201718. PMC10074084 36698303

[ref26] Chen L.; Li Y.; Li S.-P.; Fan L.-Z.; Nan C.-W.; Goodenough J. B. PEO/garnet composite electrolytes for solid-state lithium batteries: From “ceramic-in-polymer” to “polymer-in-ceramic”. Nano Energy 2018, 46, 176–184. 10.1016/j.nanoen.2017.12.037.

[ref27] Li Y.; Wang H. Composite Solid Electrolytes with NASICON-Type LATP and PVdF–HFP for Solid-State Lithium Batteries. Ind. Eng. Chem. Res. 2021, 60 (3), 1494–1500. 10.1021/acs.iecr.0c05075.

[ref28] Liu L.; Zhang D.; Zhao J.; Shen J.; Li F.; Yang Y.; Liu Z.; He W.; Zhao W.; Liu J. Synergistic Effect of Lithium Salts with Fillers and Solvents in Composite Electrolytes for Superior Room-Temperature Solid-State Lithium Batteries. ACS Appl. Energy Mater. 2022, 5 (2), 2484–2494. 10.1021/acsaem.1c04001.

[ref29] Chen S.-Y.; Hsieh C.-T.; Zhang R.-S.; Mohanty D.; Gandomi Y. A.; Hung I. M. Hybrid solid state electrolytes blending NASICON-type Li_1+x_Al_x_Ti_2–x_(PO_4_)_3_ with poly(vinylidene fluoride-co-hexafluoropropene) for lithium metal batteries. Electrochim. Acta 2022, 427, 140903 10.1016/j.electacta.2022.140903.

[ref30] Zagórski J.; López del Amo J. M.; Cordill M. J.; Aguesse F.; Buannic L.; Llordés A. Garnet–Polymer Composite Electrolytes: New Insights on Local Li-Ion Dynamics and Electrodeposition Stability with Li Metal Anodes. ACS Appl. Energy Mater. 2019, 2 (3), 1734–1746. 10.1021/acsaem.8b01850.

[ref31] Foran G.; Mery A.; Bertrand M.; Rousselot S.; Lepage D.; Aymé-Perrot D.; Dollé M. NMR Study of Lithium Transport in Liquid–Ceramic Hybrid Solid Composite Electrolytes. ACS Appl. Mater. Interfaces 2022, 14 (38), 43226–43236. 10.1021/acsami.2c10666. 36123320

[ref32] Méry A.; Rousselot S.; Lepage D.; Aymé-Perrot D.; Dollé M. Limiting Factors Affecting the Ionic Conductivities of LATP/Polymer Hybrid Electrolytes. Batteries 2023, 9 (2), 87 10.3390/batteries9020087.

[ref33] Isaac J. A.; Devaux D.; Bouchet R. Dense inorganic electrolyte particles as a lever to promote composite electrolyte conductivity. Nat. Mater. 2022, 21 (12), 1412–1418. 10.1038/s41563-022-01343-w. 36109675

[ref34] Zheng J.; Tang M.; Hu Y.-Y. Lithium Ion Pathway within Li_7_La_3_Zr_2_O_12_-Polyethylene Oxide Composite Electrolytes. Angew. Chem., Int. Ed. 2016, 55 (40), 12538–12542. 10.1002/anie.201607539. 27611222

[ref35] Zheng J.; Hu Y.-Y. New Insights into the Compositional Dependence of Li-Ion Transport in Polymer–Ceramic Composite Electrolytes. ACS Appl. Mater. Interfaces 2018, 10 (4), 4113–4120. 10.1021/acsami.7b17301. 29303244

[ref36] Yang T.; Zheng J.; Cheng Q.; Hu Y.-Y.; Chan C. K. Composite Polymer Electrolytes with Li_7_La_3_Zr_2_O_12_ Garnet-Type Nanowires as Ceramic Fillers: Mechanism of Conductivity Enhancement and Role of Doping and Morphology. ACS Appl. Mater. Interfaces 2017, 9 (26), 21773–21780. 10.1021/acsami.7b03806. 28598143

[ref37] Isaac J. A.; Mangani L. R.; Devaux D.; Bouchet R. Electrochemical Impedance Spectroscopy of PEO-LATP Model Multilayers: Ionic Charge Transport and Transfer. ACS Appl. Mater. Interfaces 2022, 14 (11), 13158–13168. 10.1021/acsami.1c19235. 35258942 PMC8949763

[ref38] Abe T.; Sagane F.; Ohtsuka M.; Iriyama Y.; Ogumi Z. Lithium-Ion Transfer at the Interface Between Lithium-Ion Conductive Ceramic Electrolyte and Liquid Electrolyte–A Key to Enhancing the Rate Capability of Lithium-Ion Batteries. J. Electrochem. Soc. 2005, 152 (11), A2151 10.1149/1.2042907.

[ref39] Vivek J. P.; Meddings N.; Garcia-Araez N. Negating the Interfacial Resistance between Solid and Liquid Electrolytes for Next-Generation Lithium Batteries. ACS Appl. Mater. Interfaces 2022, 14 (1), 633–646. 10.1021/acsami.1c17247. 34962750

[ref40] Simon F. J.; Hanauer M.; Henss A.; Richter F. H.; Janek J. Properties of the Interphase Formed between Argyrodite-Type Li_6_PS_5_Cl and Polymer-Based PEO10:LiTFSI. ACS Appl. Mater. Interfaces 2019, 11 (45), 42186–42196. 10.1021/acsami.9b14506. 31613597

[ref41] McOwen D. W.; Seo D. M.; Borodin O.; Vatamanu J.; Boyle P. D.; Henderson W. A. Concentrated electrolytes: decrypting electrolyte properties and reassessing Al corrosion mechanisms. Energy Environ. Sci. 2014, 7 (1), 416–426. 10.1039/C3EE42351D.

[ref42] Kim H.-K.; Barai P.; Chavan K.; Srinivasan V. Transport and mechanical behavior in PEO-LLZO composite electrolytes. J. Solid State Electrochem. 2022, 26 (9), 2059–2075. 10.1007/s10008-022-05231-w.

[ref43] Dong Z.; Xu C.; Wu Y.; Tang W.; Song S.; Yao J.; Huang Z.; Wen Z.; Lu L.; Hu N. Dual Substitution and Spark Plasma Sintering to Improve Ionic Conductivity of Garnet Li_7_La_3_Zr_2_O_12_. Nanomaterials 2019, 9 (5), 721 10.3390/nano9050721. 31083313 PMC6566816

[ref44] Kremer F.; Schönhals A. Broadband dielectric spectroscopy; Springer-Verlag: Berlin, Heidelberg, 2003.

[ref45] Mei W.; Yu D.; Madsen L. A.; Hickey R. J.; Colby R. H. Ion States Impact Charge Transport and Dielectric Constant for Poly(ethylene oxide)-Based Sulfonylimide Lithium Ionomers. Macromolecules 2023, 56 (13), 5141–5151. 10.1021/acs.macromol.3c00294.

[ref46] Popov I.; Cheng S.; Sokolov A. P., Broadband Dielectric Spectroscopy and Its Application in Polymeric Materials. Macromolecular Engineering: From Precise Synthesis to Macroscopic Materials and Applications; Wiley-VCH Gmbh, 2022; pp 1–39.

[ref47] Rettenwander D.; Welzl A.; Pristat S.; Tietz F.; Taibl S.; Redhammer G. J.; Fleig J. A microcontact impedance study on NASICON-type Li_1+x_Al_x_Ti_2–x_(PO_4_)_3_ (0 ≤ *x* ≤ 0.5) single crystals. J. Mater. Chem. A 2016, 4 (4), 1506–1513. 10.1039/C5TA08545D.

[ref48] Breuer S.; Prutsch D.; Ma Q.; Epp V.; Preishuber-Pflügl F.; Tietz F.; Wilkening M. Separating bulk from grain boundary Li ion conductivity in the sol–gel prepared solid electrolyte Li_1.5_Al_0.5_Ti_1.5_(PO_4_)_3_. J. Mater. Chem. A 2015, 3 (42), 21343–21350. 10.1039/C5TA06379E.

[ref49] Boaretto N.; Ghorbanzade P.; Perez-Furundarena H.; Meabe L.; López del Amo J. M.; Gunathilaka I. E.; Forsyth M.; Schuhmacher J.; Roters A.; Krachkovskiy S.; Guerfi A.; Armand M.; Martinez-Ibañez M. Transport Properties and Local Ions Dynamics in LATP-Based Hybrid Solid Electrolytes. Small 2024, 20 (10), 2305769 10.1002/smll.202305769. 37875738

[ref50] Kaufman L. A.; Huang T.-Y.; Lee D.; McCloskey B. D. Particle Surface Cracking Is Correlated with Gas Evolution in High-Ni Li-Ion Cathode Materials. ACS Appl. Mater. Interfaces 2022, 14 (35), 39959–39964. 10.1021/acsami.2c09194. 36017882

