# Supplementary material for: Lithium-Ion Transport and Exchange between Phases in a Concentrated Liquid Electrolyte Containing Lithium-Ion-Conducting Inorganic Particles
Source: ACS Energy Lett. 2024 Mar 25;9(4):1717–24. doi: 10.1021/acsenergylett.4c00502 (PMC11019636; doi:10.1021/acsenergylett.4c00502)
Supplement: Supplementary file 1 — nz4c00502_si_001.pdf [file nz4c00502_si_001.pdf]

Supporting Information for

Lithium-Ion Transport and Exchange between Phases in a Concentrated Liquid Electrolyte Containing Lithium-Ion-Conducting Inorganic Particles

Deyang Yu,[1] Zachary C. Tronstad,[1,2] Bryan D. McCloskey[1,2,*]

[1]Energy Storage and Distributed Resources Division, Lawrence Berkeley National Laboratory, Berkeley, California 94720, United States

[2]Department of Chemical & Biomolecular Engineering, University of California, Berkeley, California 94720, United States

Corresponding Author: Bryan D. McCloskey (bmcclosk@berkeley.edu)

Experimental Section

Materials. Ethylene carbonate (EC) and lithium bis(trifluoromethylsulfonyl)imide (LiTFSI) are purchased from Gotion and stored in an Ar-filled glovebox before use. Ampcera® Li_6PS_5Cl particles are purchased from MSE supplies and transferred to an Ar-filled glovebox immediately after received. All the other inorganic electrolyte particles, purchased from MSE supplies, Toshima Manufacturing, and Ohara, are dried under vacuum at 140 °C before transferring to the glovebox. The high concentration liquid electrolyte is prepared by mixing EC and LiTFSI in a 2:1 molar ratio at 80 °C. The density of the high concentration electrolyte at 25 °C is 1.66 g cm^{-3}.[1] The suspensions are prepared by mixing the high concentration electrolyte and inorganic electrolyte particles at 70 °C for 30 minutes on a hot plate under stirring. Due to the high viscosity, the suspension samples are stable for at least several hours without any noticeable particle settlement, allowing a sufficient time window to measure their conductivities. When calculating the volume fraction of inorganic particles, the following densities are used. LATP: 2.92 g cm^{-3} (from vendor), LPSCl: 1.64 g cm^{-3} (from vendor), LLTO: 5.04 g cm^{-3},[2] Ta-LLZO: 5.35 g cm^{-3},[3-4] LICGC: 2.8 g cm^{-3} (from vendor).

Characterization methods. Electrochemical impedance spectroscopy (EIS) measurements are performed on a BioLogic VMP-3 potentiostat. A lab-built cell with two Pd electrodes separated by 2.2 ± 0.05 cm is used for all the EIS measurements. The Pd wires are insulated with PTFE tubes and only the spiral ends are in direct contact with the sample. The cell constant of the lab-built cell is calibrated to be 19.5 cm^{-1} using a KCl standard solution following the procedures reported in a previous publication.[5] The impedance spectra of the cell filled with 0.1 mol kg^{-1} KCl is measured from 10 °C to 50 °C at 5 °C intervals. The conductivity of the standard solution at each corresponding temperature is obtained from literature.[6] The cell constant is then obtained from a linear fit to the plot of conductivity vs the inverse of bulk resistance. The applied AC amplitude ranges from 50 mV to 300 mV depending on the temperature and sample.

When measuring EIS at low temperatures (e.g. 10 °C), a 300 mV AC amplitude is necessary in order to obtain smooth Nyquist plots due to the large resistance of the samples at this temperature. Although a 300 mV AC amplitude seems very high compared to common EIS measurements (10 mV), the large distance between Pd electrodes ensures a weak electric field strength. The applied frequency range for all the measurements is 1 MHz − 0.1 Hz, with 20 data points for each decade. The first five data points were deleted after performing a Kramers-Kronig validation.[7-9] EIS data fit was conducted using the BioLogic EC-Lab® V11.43 software. The overall resistance, which is determined from the intersection of the low-frequency straight line and the semicircle in the Nyquist plots, are used to calculate the conductivity of suspension samples according to equation (1). The overall resistance agrees very well with the sum of R1 and R2 obtained from EIS fitting.

Diffusion coefficients are measured using pulsed-field-gradient nuclear magnetic resonance (PFG-NMR) on a Bruker AvanceNEO 400 MHz spectrometer coupled with a diffBB probe at 25 °C using the pulsed-gradient double stimulated-echo sequence (PGDSTE) in a similar way as previously reported.[10] The diffusion time (Δ) was set to 100 ms, and the maximum gradient was set to ≤ 1450 gauss cm^{-1}. Diffusion coefficients of EC at Δ = 20 ms and 500 ms were also measured, and the results shows negligible change (< 5 %). When measuring the electrophoretic mobility of Li^+ in the high concentration liquid electrolyte EC/LiTFSI=2/1, 0.5 wt% poly(ethylene oxide) (M_w 600k) is added to the liquid electrolyte to suppress convection. Electrophoretic NMR is conducted in a similar way as previously reported with an effective gradient pulse duration of 3.0 ms, a Δ of 100 ms, a gradient of 1200 gauss cm^{-1}, and an electric field strength of 0 ~ ±125 V cm^{-1}.[5,11-12]

For the Li^+/Na^+ exchange experiment, a 10 wt% $NaClO_4$ in EC solution was first prepared in a glovebox. Then 134 mg LATP particles and 560 mg $NaClO_4$/EC solution (0.4 mL) was quickly mixed and stirred at 30 °C on a hot plate. After a certain mixing time, the mixture was filtered using a 0.2 μm PTFE

Whatman™ syringe filter. The filtered solution was clear without any noticeable particles. The filtered solution was filled into a 5 mm NMR tube. A 3 mm NMR tube filled with an external standard, an acetone solution containing 0.5 M $NaClO_4$ and 0.5 M LiTFSI, was inserted into the 5 mm NMR tube. The height of the sample and standard solution were over 3 cm, sufficient to cover the whole NMR coil region. The ^{23}Na and ^{7}Li NMR spectra was recorded using a Bruker Ascend™ 500 MHz spectrometer at ambient temperature with a 90° pulse. The recycle delay (d1) was set to > 5 × T_1. The concentration of Na^+ and Li^+ in the sample can be calculated by measuring the geometry of the 3 mm and 5 mm tubes. The molar ratio between Na^+ and Li^+, which is irrelevant to the NMR tube geometries, can be further calculated, or obtained directly from the NMR peak integrals.

Table S1. Fitting parameters for the Nyquist plots shown in Figure 1c and the characteristic frequencies for R1 and R2 processes.

LATP weight content	LATP volume content	C (F)	R1 (kΩ)	Q (F $s^{\alpha-1}$)	α	R2 (kΩ)	$\frac{1}{2\pi\,R1\,C}$ (kHz)	$\frac{1}{2\pi\,(R2\,Q)^{1/\alpha}}$ (kHz)
0 %	0.0 %	4.59×10^{-11}	118	NA	NA	NA	29.4	NA
10 %	5.9 %	5.87×10^{-11}	83.2	1.28×10^{-9}	0.907	31.5	32.6	11.2
20 %	12.4 %	5.93×10^{-11}	61.2	8.85×10^{-10}	0.893	48.8	43.9	12.3
30 %	19.6 %	6.67×10^{-11}	45.2	9.89×10^{-10}	0.865	61.4	52.7	11.9
40 %	27.5 %	6.00×10^{-11}	36.9	1.17×10^{-9}	0.840	71.3	72.0	11.5
50 %	36.2 %	6.70×10^{-11}	27.8	1.31×10^{-9}	0.827	83.2	85.5	9.86

Note: the high frequency semicircle is fitted with a parallel resistor R1 and a capacitor, and the characteristic frequency is expressed as $\frac{1}{2\pi\,R1\,C}$ in Hz. The sunken semicircle at medium frequency is fitted with a resistor R2 in parallel with a constant phase element Q, whose impedance is $\frac{1}{Q(i\omega)^{\alpha}}$. Thus, the overall impedance expression of the parallel R2 and Q is $Z^{*} = \frac{R2}{1 + R2\,Q\,(i\omega)^{\alpha}}$. Comparing this equation with the Cole-Cole equation for complex dielectric relaxation $\frac{\varepsilon^{*}-\varepsilon_{\infty}}{\Delta\varepsilon} = \frac{1}{1+(i\omega\tau_{cc})^{\beta}}$, we can define $(R2\ Q)^{1/\alpha}$ as a characteristic time, and thus the characteristic frequency in Hz would be $\frac{1}{2\pi\,(R2\,Q)^{1/\alpha}}$.

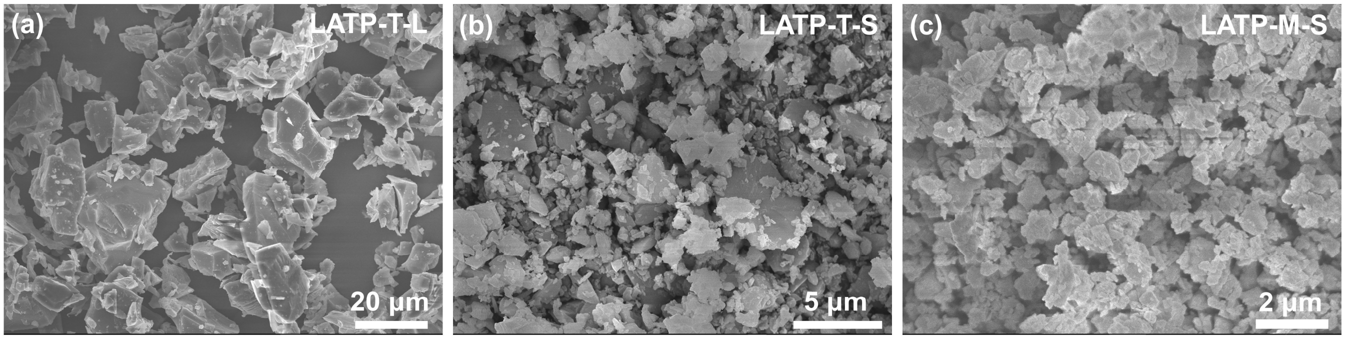

Figure S1. SEM images of LATP-T-L (**a**), LATP-T-S (**b**), and LATP-M-S (**c**). The particle size of LATP-T-L is substantially larger than the other two samples. LATP-T-S contains a small fraction of large particles with a size of a few microns, while the particle size of LATP-M-S is more homogeneous.

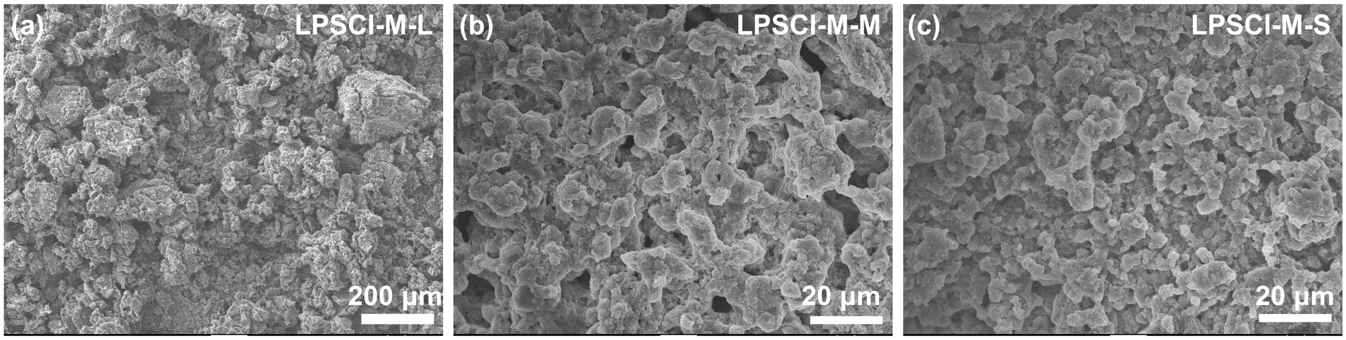

Figure S2. SEM images of LPSCl-M-L coarse powder (**a**), LPSCl-M-M fine powder (**b**), and LPSCl-M-S ultra-fine powder (**c**). The connection between particles in LPSCl-M-M and LPSCl-M-S could be caused by moisture when the samples are shortly exposed to air during the sample preparation process.

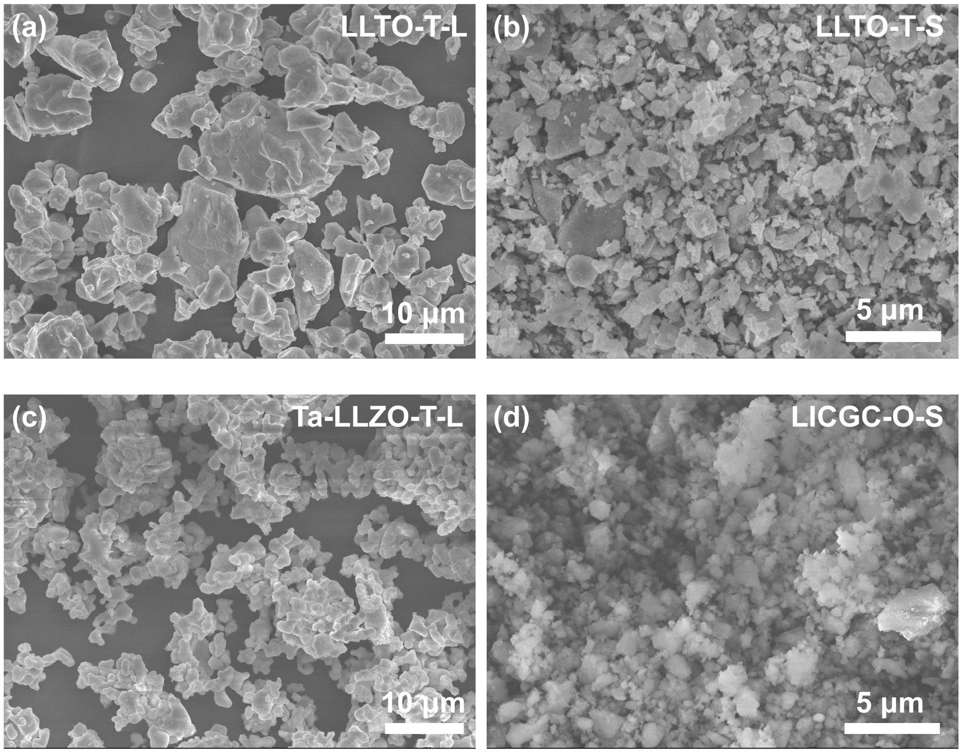

Figure S3. SEM images of LLTO-T-L (a), LLTO-T-S (b), Ta-LLZO-T-L (c), and LICGC-O-S (d). The particle size of LLTO-T-L is substantially larger than LLTO-T-S. The primary particles in Ta-LLZO-T-L with a size of a few microns form larger aggregates. The particle size of LICGC-O-S is ~ 1 μm.

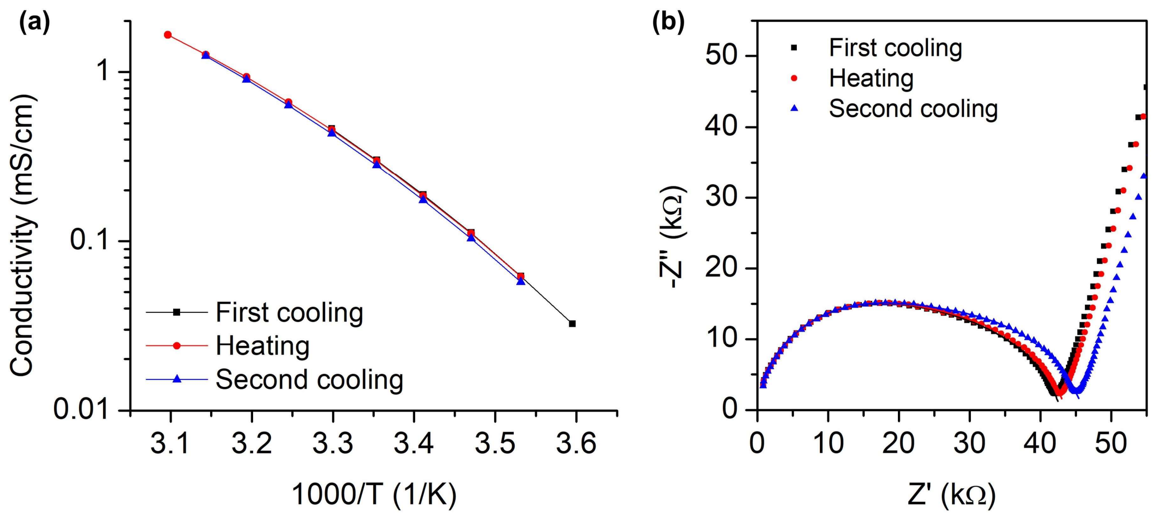

Figure S4. Stability test of the impedance measurements. The suspension containing 36.3 vol% LPSCl-M-L undergoes a cooling-heating-cooling cycle. The sample is cooled from 30 °C to 5 °C, followed by heating to 50 °C, and finally another cooling process to 10 °C. Impedance spectra are recorded at 5 °C intervals. It takes about 0.5 hour for the impedance measurement at each temperature, including the time needed for temperature equilibrium. This cooling-heating-cooling cycle takes a total of 12.5 hours. (**a**) Arrhenius plot of the overall conductivity, calculated from the intersection of the medium-frequency semicircle and low-frequency line. (**b**) Impedance spectra at 30 °C for the first cooling process (black square), the heating process (red circle), and the second cooling process (blue triangle). The increase in the total resistance from the initial 30 °C to the second cooling process is about 7%, which could be caused by particle precipitation and the side reactions between LPSCl and the liquid electrolyte.

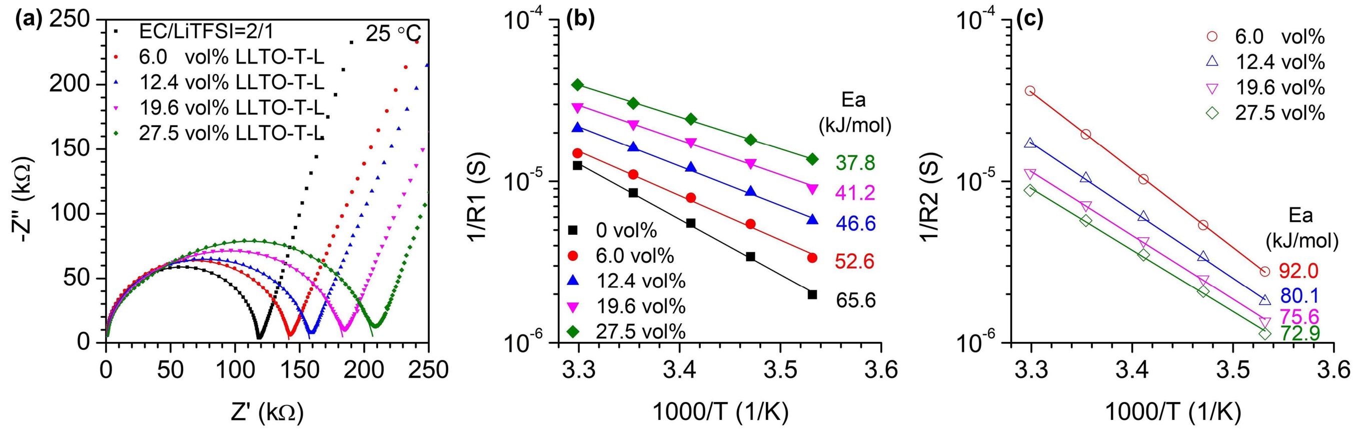

Figure S5. (**a**) Nyquist plots of LLTO-T-L particles suspended in EC/LiTFSI=2/1 at 25 °C. Temperature dependence of 1/R1 (**b**) and 1/R2 (**c**) from 10 °C to 30 °C.

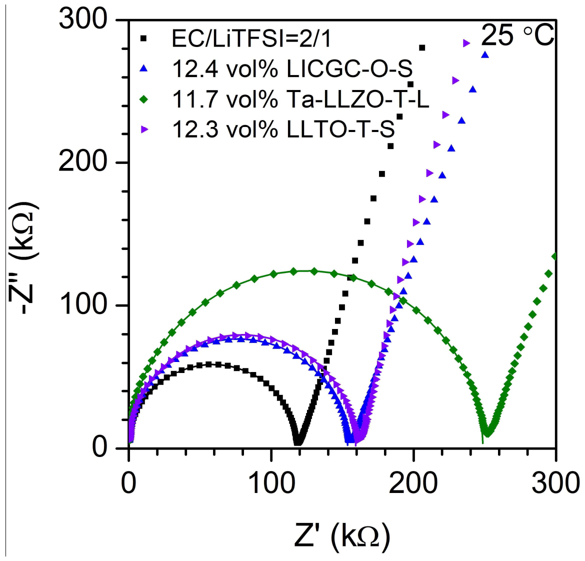

Figure S6. Nyquist plots of EC/LiTFSI=2/1 and the suspensions with LICGC-O-S, Ta-LLZO-T-L, and LLTO-T-S at 25 °C.

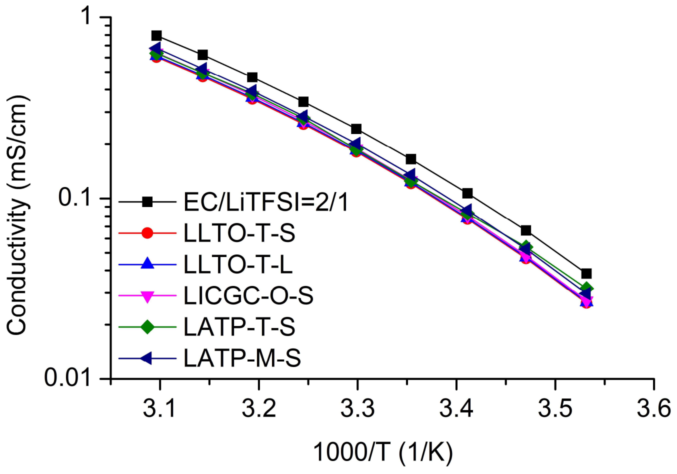

Figure S7. Ionic conductivities of neat EC/LiTFSI=2/1 and the suspensions with 12 vol% various inorganic electrolyte particles from 10 °C to 50 °C. The conductivities of suspensions with the same volume fraction of LLTO-T-S, LLTO-T-L, LICGC-O-S, LATP-T-S, and LATP-M-S are very close to each other, indicating that these materials contribute little to ion transport.

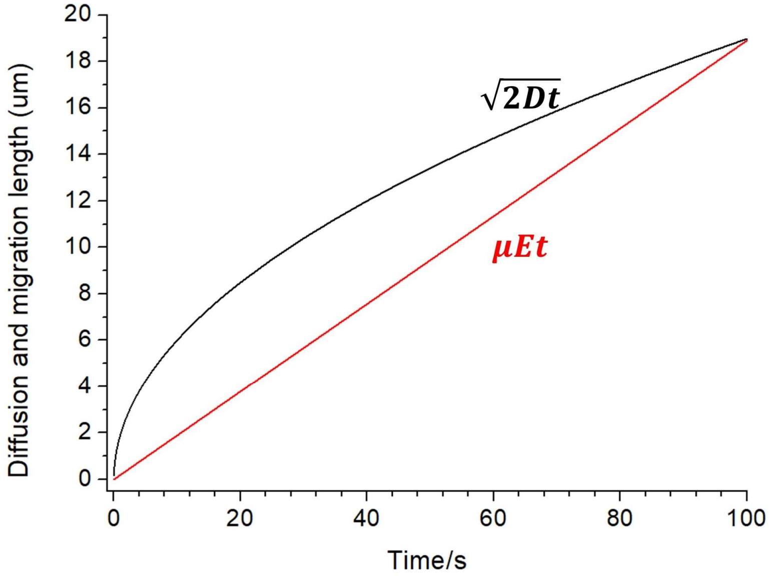

Figure S8. A comparison of the one-dimentional diffusion length ($\sqrt{2Dt}$) and migration length (μEt) of Li^+ in the EC/LiTFSI=2/1 electrolyte. The self-diffusion coefficient (D) and mobility (μ) of Li^+ is 1.8×10^{-12} m^2 s^{-1} and 3.8×10^{-11} m^2 s^{-1} V^{-1}, respectively at 30 °C. An electric field strength (E) of 5×10^3 V m^{-1} (0.1V

voltage drop over 20 μm) is used to calculate the migration length. This comparison clearly indicates that the motion of Li^+ in the liquid electrolyte is dominated by diffusion on a length scale of 1 μm, making its motion looking like a random walk.

References:

(1) Nilsson, V.; Kotronia, A.; Lacey, M.; Edström, K.; Johansson, P. Highly Concentrated LiTFSI–EC Electrolytes for Lithium Metal Batteries. *ACS Appl. Energy Mater.* **2020,** *3* (1), 200-207.

(2) Cho, Y.-H.; Wolfenstine, J.; Rangasamy, E.; Kim, H.; Choe, H.; Sakamoto, J. Mechanical properties of the solid Li-ion conducting electrolyte: Li0.33La0.57TiO3. *Journal of Materials Science* **2012,** *47* (16), 5970-5977.

(3) Kataoka, K.; Akimoto, J. Lithium-ion conductivity and crystal structure of garnet-type solid electrolyte Li_{7−}<i>_x</i>La₃Zr_{2−}<i>_x</i>Ta<i>_x </i>O₁₂ using single-crystal. *J. Ceram. Soc. Jpn.* **2019,** *127* (8), 521-526.

(4) Dong, Z.; Xu, C.; Wu, Y.; Tang, W.; Song, S.; Yao, J.; Huang, Z.; Wen, Z.; Lu, L.; Hu, N. Dual Substitution and Spark Plasma Sintering to Improve Ionic Conductivity of Garnet Li7La3Zr2O12. *Nanomaterials* **2019,** *9* (5), 721.

(5) Yu, D.; Troya, D.; Korovich, A. G.; Bostwick, J. E.; Colby, R. H.; Madsen, L. A. Uncorrelated Lithium-Ion Hopping in a Dynamic Solvent–Anion Network. *ACS Energy Letters* **2023**, 1944-1951.

(6) Wu, Y. C.; Koch, W. F.; Pratt, K. W. Proposed New Electrolytic Conductivity Primary Standards for KCl Solutions. *J. Res. Natl. Inst. Stand. Technol.* **1991,** *96* (2), 191-201.

(7) Schönleber, M.; Klotz, D.; Ivers-Tiffée, E. A Method for Improving the Robustness of linear Kramers-Kronig Validity Tests. *Electrochim. Acta* **2014,** *131*, 20-27.

(8) Boukamp, B. A. A Linear Kronig ‐ Kramers Transform Test for Immittance Data Validation. *J. Electrochem. Soc.* **1995,** *142* (6), 1885.

(9) https://www.iam.kit.edu/et/english/Lin-KK.php (accessed 2023-11-23).

(10) Yu, D.; Pan, X.; Bostwick, J. E.; Zanelotti, C. J.; Mu, L.; Colby, R. H.; Lin, F.; Madsen, L. A. Room Temperature to 150 °C Lithium Metal Batteries Enabled by a Rigid Molecular Ionic Composite Electrolyte. *Adv. Energy Mater.* **2021,** *11* (12), 2003559.

(11) Yu, D.; Min, J.; Lin, F.; Madsen, L. A. Mechanically and Thermally Robust Gel Electrolytes Built from A Charged Double Helical Polymer. *Adv. Mater.* **2024**, https://doi.org/10.1002/adma.202312513.

(12) Bergstrom, H. K.; McCloskey, B. D. Ion Transport in (Localized) High Concentration Electrolytes for Li-Based Batteries. *ACS Energy Letters* **2024**, 373-380.
